# Narrative Abilities of Adults’ With Down Syndrome as a Window to Their Morphosyntactic, Socio-Cognitive, and Prosodic Abilities

**DOI:** 10.3389/fpsyg.2020.02060

**Published:** 2020-08-26

**Authors:** Maria Martzoukou, Anastasia Nousia, Theodoros Marinis

**Affiliations:** ^1^School of Health Sciences, University of Ioannina, Ioannina, Greece; ^2^Department of Linguistics, University of Konstanz, Konstanz, Germany; ^3^School of Psychology and Clinical Language Sciences, University of Reading, Reading, United Kingdom

**Keywords:** down syndrome, microstructure, macrostructure, prosody, narratives, Theory of Mind

## Abstract

Down syndrome (DS) is the most common developmental disorder characterized by mild to moderate intellectual disability. Several studies have reported poor language and prosodic skills and contradictory results regarding individuals’ with DS socio-cognitive skills, whereas most of them have focused on children with DS. The present study attempts to explore adults’ with DS language, socio-cognitive and prosodic abilities via the use of story-retellings. Twenty adults with DS and two groups of TD children, one matched to their expressive vocabulary (TD-EVT) and the other matched to their non-verbal mental age (TD-RCPM), took part in the present study. Participants listened to a story while viewing a wordless picture PowerPoint presentation on a computer screen, and then, they were instructed to retell the story while viewing the pictures for a second time. Each participant listened to two stories, one with “lively” and one with “flat” prosody. Results revealed that adults’ with DS performance was comparable with the one presented by the TD-RCPM group, whereas the TD-EVT group performed significantly better in almost all variables. Individuals’ with DS re-narrations, however, contained significantly less complement clauses and internal state terms (related or not related to Theory of Mind–ToM) compared to the re-narrations of both control groups. In contrast, the group with DS performed similarly to both control groups in comprehension questions related to main characters’ internal state terms and significantly better compared to the TD-RCPM group in questions related to ToM. In terms of prosody, all three groups performed significantly better on story structure and comprehension questions when prosody was “lively” compared “flat” prosody. DS group’s re-narrations did not contain enough internal state terms, not due to their inability in recognizing them, but due to their poor morphosyntactic abilities, which did not allow them to find the proper means to express the main characters’ internal states. Prosody facilitated participants with DS in the comprehension and re-narration. This suggests that intervention programs based on prosody could support the language skills of adults with DS.

## Introduction

Down syndrome (DS) is the most common developmental disorder causing mild to moderate intellectual disability (Loane et al., 2013; Presson et al., 2013). In most cases (95%), DS results by the presence of a full third copy of chromosome 21 (trisomy), while in the rest of the cases the third copy occurs only in some cells (mosaicism) or parts of chromosome 21 are attached to another chromosome (Martin et al., 2009). Although wide individual variation has been noted, depending on the experimental procedures used and the age of the participants studied, the main characteristics of DS phenotype are: later onset and slower pace of language development, better language comprehension than production abilities, particularly impaired morphosyntax, relative strength in vocabulary, especially the receptive one, and poor phonological working memory compared to visual spatial memory (Fowler, 1990; Chapman et al., 1998; Miller, 1999; Chapman and Hesketh, 2000; Abbeduto et al., 2003, 2007; Iverson et al., 2003; Laws and Bishop, 2003; Miolo et al., 2005; Ypsilanti et al., 2005; Martin et al., 2009; Finestack and Abbeduto, 2010; Finestack et al., 2013; Phillips et al., 2014).

Speech production of individuals with DS is characterized by substitutions, omissions and inconsistencies of speech sounds (e.g., Dodd, 1976; Chapman and Hesketh, 2001). According to some researchers (e.g., Bray et al., 1995; Heselwood et al., 1995), such phonological problems are associated with individuals’ with DS difficulties in identifying syllable, word and phrase boundaries, due to their poor prosody comprehension abilities. The aforementioned claim is in line with the Perceptual Salience Approach (Echols and Newport, 1992), according to which prosody facilitates the development of phonological identification and awareness, in that syllables that are perceptually salient (e.g., marked by a higher pitch and longer duration) are comprehended and produced in greater phonological detail compared to less salient syllables (Grosjean and Gee, 1987; Echols and Newport, 1992; Beattie and Manis, 2014). Therefore, impaired prosody comprehension abilities lead to poor phonological awareness and erroneous speech production. Despite these claims, most studies dealing with prosody in individuals with DS have focused on prosody production rather than comprehension. The results demonstrate prosodic abnormalities mainly on frequency, duration and intensity, which are attributed to physiological peculiarities, such as smaller vocal tract compared to the size of the tongue, soft palatal shape and muscular hypotonia (e.g., Lee et al., 2009; Albertini et al., 2010; Kent and Vorperian, 2013; Rochet-Capellan and Dohen, 2015; Corrales-Astorgano et al., 2016, 2018). In contrast to the large number of production studies, very few studies have explored the perception abilities of prosody in children and adolescents with DS (Pettinato and Verhoeven, 2009; Stojanovik, 2011; Naess, 2016). The results of these studies revealed difficulties in word stress processing (Pettinato and Verhoeven, 2009), weaker phonological awareness and delayed awareness of rhyme compared to non-verbal mental age matched controls (Naess, 2016), and good performance on discriminating sounds when compared to non-verbal mental age matched controls, but poor when compared to chronological age matched controls (Stojanovik, 2011). Thus, to date a small number of aspects of prosody have been explored only in children and adolescents and the results are not always consistent. Therefore, more research in clearly needed, especially in adults with DS, for which there are no studies focusing on their production and perception of prosody. New research findings might also shed light to the ongoing debate regarding whether prosody is independent of general cognitive impairments (Wells and Peppé, 2003).

Morphosyntax is another aspect of language which seems to cause problems to individuals with DS (e.g., Martin et al., 2009). In particular, difficulties have been reported in the comprehension of inflectional and derivational morphology (e.g., plural –s, and past tense –ed) (e.g., Abbeduto et al., 2003; Price et al., 2007) and of complex sentences (e.g., passives, negatives, questions) (e.g., Rosin et al., 1988; Abbeduto et al., 2003; Joffe and Varlokosta, 2007; Price et al., 2007; Caselli et al., 2008). Even though the comprehension of morphosyntax seems to cause difficulties to individuals with DS, their production ability is more profoundly impaired. Participants’ with DS productions have been found to be characterized by omissions or errors in the use of past tense –ed, present progressive –ing, third-person singular -s, modals and articles (Fowler et al., 1994; Hesketh and Chapman, 1998; Eadie et al., 2002; Ring and Clahsen, 2005; Caselli et al., 2008) and by different preferences and strategy adoptions while marking the past tense in novel verbs (Stathopoulou and Clahsen, 2010). Moreover, they have been reported to produce shorter and less complex sentences compared to typically developing children matched for non-verbal mental age (Rosin et al., 1988; Chapman et al., 1998; Caselli et al., 2008; Price et al., 2008).

Lastly, compared to their language skills, far less is known about the socio-cognitive abilities of individuals with DS, namely the cognitive processes involved during social interactions (Frith and Frith, 2007). The stereotypical perceptions, according to which individuals with DS are highly sociable (Down, 1866), led to the assumption that their socio-cognitive skills are relatively intact and, consequently, to a paucity of research regarding this issue (Cebula et al., 2010). The existing limited literature has mainly focused on the comparison between children with DS and children with other developmental disorders, such as autism, and the results revealed relatively well-preserved communicative strategies and Theory of Mind (ToM) skills, namely the understanding of the mental states of others and the use of this information to predict others’ behavior (Abbeduto and Chapman, 2005; Roberts et al., 2007; Fidler et al., 2008; Klusek et al., 2014; Martin et al., 2013). However, when compared to typically developing (TD) mental age-matched controls, children with DS are reported to demonstrate socio-cognitive impairments, such as introducing fewer new topics (Abbeduto et al., 2008), facing difficulties in providing adequate background when introducing new topics (Lee et al., 2017), using inappropriate initiation of conversations, having difficulties in understanding the context, using phrases in inappropriate contexts (Smith et al., 2017) and increased use of stereotyped language (Laws and Bishop, 2004) (for a review see also Wishart, 2007). As far as the socio-cognitive abilities of adults with DS are concerned, research is limited on exploring their ability to recognize facial expressions of emotions and their perception of friendship. For example, Carvajal et al. (2012) found no statistical significant difference between adults with DS and adults with other intellectual disabilities on their ability to recognize emotions from faces. On the other hand, Virji-Babul et al. (2012) found statistical significant differences, when adults’ with DS performance was compared to that of TD adults, but poor performance on the recognition of scared faces when adults with DS were compared to TD children matched on mental age. Regarding the perception of friendship, adults with DS made more errors in identifying “friends” from “non-friends” but they were found to be equally able to distinguish friendly behaviors and actions from non-friendly behaviors as their chorological age and mental age matched peers.

A more spherical view of the outcomes of the aforementioned studies, though, reveals that individuals’ with DS deficits in socio-cognitive abilities are more pronounced when a concurrent verbal task is used (see also Reed and Steed, 2015 for a connection between language and the understanding of emotions). This observation is in line with the Construction Hypothesis (Lindquist and Gendron, 2013), according to which language is of major importance in emotion perception, since one should be able to understand the exact meaning of a word (e.g., anger) and the behaviors and context to which it is linked. Pochon and Declercq (2013, 2014) and Cebula et al. (2017) claim that difficulties found in individuals with DS in emotion recognition tasks are due to their inability to connect emotion labels with the relevant emotion presented by pictures or vocal stimuli. Thus, they conclude that individuals with DS have a specific emotional lexicon deficit rather than a difficulty in recognizing emotional expression. Thus, emotional lexicon could be responsible for their inability to label someone as a “friend” or “non-friend” or a face as depicting fear (Virji-Babul et al., 2012), as well as their ability to follow the conversational conventions by using the appropriate language means (Laws and Bishop, 2004; Wishart, 2007; Abbeduto et al., 2008; Lee et al., 2017; Smith et al., 2017). The contradictory results and the under-investigated theories combined with the fact that most of the aforementioned studies were focused on children’s socio-cognitive skills make it clear that more research is needed, especially regarding socio-cognitive abilities in adults with DS.

One ecological way to investigate individuals’ linguistic and socio-cognitive skills is by using narrative tasks. Narrative ability is the ability to generate or reproduce a personal or fictional story usually by referring to past events and by presenting ones’ own and others’ perspectives (Boudreau and Chapman, 2000; Miles and Chapman, 2002; Finestack et al., 2012; Channell et al., 2015; Ashby et al., 2017). Narratives can be evaluated at two levels: microstructure and macrostructure. Microstructure refers to the linguistic structures used by the narrator and is mainly focused on mean length of utterance (MLU) in morphemes or words, number of different content or function words, and number of main or dependent clauses. On the other hand, macrostructure focuses on the socio-cognitive use of language and, in particular, on the narrator’s ability to communicate the most relevant information regarding the context, the episodes of the story, as well as the characters and their perspectives (actions, reactions, and intentions) (e.g., Gagarina et al., 2012; Tsimpli et al., 2016; Ashby et al., 2017).

In particular, a story must include an introduction to setting and characters, initiating events, a problem, a resolution and a formal ending (Hudson and Shapiro, 1991; Cook and Guéraud, 2005; Gagarina et al., 2012; Tsimpli et al., 2016; Ashby et al., 2017). It has been argued, though, that for a successful narration of a story, especially a fictional one, the narrator must have some previous world knowledge, namely knowledge about events and things that can go wrong, personal experiences of similar occasions or memory of similar fictional stories (Hudson and Shapiro, 1991; Cook and Guéraud, 2005). Preschool children, for example, usually omit many of the basic units of a story, probably due to the lack of relevant story schemas (e.g., Mandler and DeForest, 1979; Seidman et al., 1986). Thus, world knowledge helps the development and the improvement of socio-cognitive abilities. The understanding and consideration of other people’s intentions, motivations, thoughts, and feelings consist a higher ability (ToM ability), which is not acquired before the age of 6–8 (e.g., Smith, 1978).

Therefore, the benefits of adopting a narrative task are that it allows us to explore participants’ linguistic skills, episode scaffolding, and the narrator’s ToM abilities, since presenting characters’ reactions, thoughts, and feelings is an indication of ToM ability (Lorusso et al., 2007; Tomasello, 2003; Tsimpli et al., 2016). Thus, a narrative task can give us a clear view of participants’ language and socio-cognitive abilities (including ToM), since many different variables can be taken into account and direct comparisons can be made between them.

### Narrative Production in Adults With DS

Although narrative research has mainly focused on children and adolescents with DS (for a review, see Segal and Pesco, 2015), there are some studies which also included adults with DS. Below we report some of the most recent studies in which the experimental procedure or the variables are similar or connected to the ones used in the present study. Presentation is only limited to findings related to individuals with DS and their TD counterparts.

Miles et al.’s (2006) study included 28 individuals (adolescents and young adults) with DS (age range: 13–21) and 14 TD children (age range: 3–6), matched on age-equivalent scores on syntax comprehension. Participants were examined on interview language and narrations based on wordless picture stories. The results revealed that individuals with DS had a larger MLU in narrations compared to conversations. Further analysis revealed that pictures functioned as a form of scaffolding for participants with DS which allowed them to perform similarly to the controls.

Keller-Bell and Abbeduto (2007) compared the narratives of 23 individuals with DS (age range: 13–24), 18 adolescents and young adults with fragile X syndrome (age range: 12–23), and 21 TD children matched at the group level to the two experimental groups’ mean and range of non-verbal mental age. Each participant viewed a book page by page once to become familiarized with the story, and during a second viewing, she was instructed to tell the story page by page to the experimenter. The results revealed no differences between the individuals’ with DS and the control group’s performance on their MLU in words, number of different words, and number of main and dependent clauses. Lastly, Keller-Bell and Abbeduto (2007) reported that participants with DS used a greater proportion of evaluations (mental state verbs, character names, character dialogue, repetition, sound effects, and exaggeration) in their narratives compared to their TD peers. However, as Ashby et al. (2017) report, a closer look revealed that this difference was only due to sound effects used by individuals with DS and not due to others evaluations.

Finestack et al. (2012) included overlapping participant samples that were used in several other studies, such as in Keller-Bell and Abbeduto (2007) and instructed participants to retell a story while viewing a wordless picture book for a second time. Results demonstrated that the 24 individuals with DS (age range: 12–23) performed significantly better than the TD children matched on non-verbal mental age in introducing characters and story settings (conflict/resolution and cohesion of events). Further analyses revealed that when DS participants were individually matched to controls on MLU, there was no group difference in overall scores. The participants’ with DS greater world-knowledge, due to their chronological age, helped them produce more complete stories.

Ashby et al. (2017), also drew their participants (23 with DS, 22 with fragile X syndrome and 23 TD children) from the same pool as Keller-Bell and Abbeduto (2007) and Finestack et al. (2012) and examined their use of inferential language in their narratives elicited with the use of a wordless picture book. In particular, they measured mentions of characters’ physical actions or attempts and internal states, references to causality of events, use of character dialogue and any other inference which was not directly visible on the pages of the book. Participants with DS used less inferential language compared to their TD counterparts matched on non-verbal mental age. However, when the two groups were matched on MLU in morphemes, this difference was not statistically significant for the references to character actions or attempts. The researchers questioned whether these results are due to individuals’ with DS impaired socio-cognitive ability to understand inferences or due to their poor syntactic ability which constrains them from using complex structures as the ones needed in inferential language. Comprehension questions following participants’ narrations could have given more information regarding DS participants’ understanding of inference, but no such questions were used.

Only one study (Loveland et al., 1990) contained comprehension questions after participants’ re-narrations of a puppet show or video with actors. The results showed that the 16 individuals with DS (mean age: 5–27 years old) performed significantly better compared to mental age matches with autism on comprehension questions about episodes of the story, characters’ feelings and thoughts, and speculations about implications of themes given in the story. Although the study did not include a typically developing group, the results demonstrated that individuals with DS had comprehended important aspects of the story.

As it is obvious from the above, adults’ with DS language, socio-cognitive and prosody comprehension skills remain poorly understood. Studies exploring narrative abilities in individuals with DS contain groups with a wide age range (from children to adults), while no studies have focused only on adults. Moreover, as some researchers report (e.g., Cuskelly et al., 2016; Witecy and Penke, 2017), several abilities of individuals with DS improve throughout childhood and adolescence but reach a plateau during adulthood. Having this in mind, as well as the late onset and slower pace in language development of children with DS, it is important to investigate the language and socio-cognitive plateau they reach as adults. Furthermore, most of the studies report DS participants’ performance either on microstructure or on macrostructure and they do not compare their performance on the two levels of narrations. In order to get a better understanding regarding the language and socio-cognitive abilities of individuals with DS and the connection between the two aspects, narrations should be analyzed and compared in both levels within the same individuals. In addition, in most previous studies apart from Loveland et al. (1990), participants’ story (re)narrations were not followed by comprehension questions, which can demonstrate whether participants poor performance on (re)narrations is due to language production constraints or to limited social-cognitive comprehension ability. Lastly, although prosody has been found to enhance typically developed individuals’ comprehension and verbal memory (e.g., Shintel et al., 2014), it is unclear if this is also valid for individuals with DS who are reported to have poor prosody comprehension abilities (e.g., Pettinato and Verhoeven, 2009; Naess, 2016). Such research is important because prosody comprehension research has mainly focused on children and adolescents, with contradictory results, and because it is unclear if prosody is independent of morphosyntactic and general socio-cognitive impairments.

The aim of the present study is to fill this gap by conducting a systematic examination of adults’ with DS microstructure and macrostructure abilities as well as their ability to use prosody for comprehension. In terms of the microstructure, we analyzed the use of different (a) content and (b) function words, (c) the MLU in words and (d) the types of subordination, namely total counts of adverbial, relative and verb-complement clause. In terms of the macrostructure, we addressed (e) the evaluation of the story structure according to the MAIN conventions (Gagarina et al., 2012), (f) the number of internal state terms, and (g) the ToM references in their story-retellings. To explore the participants’ (h) auditory processing, we analyzed their performance on the comprehension questions. Lastly, (i) we explored whether prosodic features, such as intonation and timbre, assist their comprehension by examining the structure story of their re-narrations and their accuracy scores on comprehension questions. By investigating the narrative abilities of adults with DS, a population which has not been systematically investigated before, the results of our study provide valuable new insights on the use of prosody for comprehension and can add to the debate regarding the relationship between morphosyntactic and socio-cognitive abilities.

## Methodology

### Participants

Twenty adults (11 females) with DS between 19 and 46 years old took part in the study. All participants were monolingual Greek speakers and had no major uncorrected physical or sensory impairments that would interfere with their ability to participate in the study. Participants with DS were recruited from “The Down Syndrome Association of Greece” and were matched to two control groups on a one to one basis. The first control group consisted of 20 typically developing (TD) children who were matched to the DS individuals on their non-verbal cognitive ability level, as evaluated using the age-equivalent scores of the Raven’s Colored Progressive Matrices (RCPM; Raven et al., 2008) (TD-RCPM) (see Table 1).

**TABLE 1:** Participants’ demographical information.

	DS (y;m)	TD-RCPM (y;m)	TD-EVT. (y;m)
Gender ratio	11F/9M	11F/9M	11F/9M
Chronological age (mean)	28;2	4;2	5;10
Chronological age (range)	18;7–45;11	3;11–6;2	3;11–10;2
Age equivalent scores (mean)		4;3	5;8
Age equivalent scores (range)		<4–6;6	3;9– 9;8
*p*-value		0.705	0.811

*DS, Down syndrome; TD-RCPM, Typically developing group matched to DS individuals on their age score equivalents on the Raven’s Colored Progressive Matrices Test; TD-EVT, Typically developing group matched to DS individuals on their age score equivalents on Expressive Vocabulary Test; y, years; m, months; F, Female; M, Male, p-value was calculated by comparing the mean age equivalent scores of the DS group on each test (RCPM and EVT) to the chronological age of the two TD groups.*

The second control group consisted of 20 TD children who were matched to individuals with DS on their verbal ability level, as evaluated using the age equivalent scores of the Expressive Vocabulary Test (EVT; Vogindroukas et al., 2009; adaptation from Renfrew, 1997) (TD-EVT) (see Table 1). The EVT assesses the children’s ability to name 50 black and white pictures. It has been supported that expressive vocabulary is closely related to morphosyntax, both by experimental studies (e.g., Miller, 1991; Ukrainetz and Blomquist, 2002; Lee, 2011; DeThrone, unpublished) and theoretical accounts (constructivist theory) according to which vocabulary and morphosyntactic development are governed by the same mechanism (Bates and Goodman, 1997, 1999, 2001). Thus, it can be argued that the measure of expressive vocabulary could give us a view of participants’ general language abilities.

The DS group was significantly older than the two groups of TD children (*p* < 0.001).

### Baseline Tasks

A battery of baseline tasks was administered prior to the main experiment to evaluate the participants’ non-verbal cognitive abilities, their expressive and receptive vocabulary, their morphosyntactic abilities, and their phonological short-term memory.

The RCPM (Raven et al., 2008) was used to assess the participants’ non-verbal cognitive abilities and the EVT (Vogindroukas et al., 2009; adaptation from Renfrew, 1997) to measure their expressive vocabulary. Since the adults’ with DS performance to RCPM and EVT was found to be similar to that expected by preschool and school-aged children, all other tasks were chosen to be appropriate for children of these ages. The participants’ receptive vocabulary ability was assessed using the Greek Peabody Picture Vocabulary Test (PPVT; Simos et al., 2011), a picture selection task.

Participants’ morpho-syntactic abilities and working memory were assessed using a Sentence Repetition test for preschool children (SRT; Stavrakaki and Tsimpli, 2000). According to Tsimpli et al. (2016), sentence repetition examines both morpho-syntactic and working memory abilities (see also Potter and Lombardi, 1998; Vinther, 2002; Riches et al., 2010). To make sure that all participants would hear the sentences in exactly the same way, all sixteen sentences had been pre-recorded by a trained female phonetician. Each sentence was presented separately and participants were instructed to repeat them verbatim. Three points were awarded for each correctly repeated sentence, two points if participants made one error, one point if they made two errors, and no points if they made more than two errors.

Phonological short-term memory was assessed using the Forward Digit Recall sub-test from the Athena Test (FDR; Paraskevopoulos et al., 1996). In this sub-test, participants were presented with a set of digits, starting with sets of 2 and increasing to sets of 7 digits. The criterion for moving on to the next set was correct recall of the set in the first or second attempt. The procedure stopped if participants failed to correctly recall a digit set twice.

### Experimental Tasks

The LITMUS-MAIN tool (Peristeri et al. in Gagarina et al., 2012) was used to assess story retelling. Two of the four stories of the tool were selected, the Baby Birds and the Dog story, each having four main characters. Each story is depicted in six colored pictures. For the needs of the present study the stories were recorded by an actress. To explore prosody comprehension, each story was recorded twice, once with “lively” and once with relatively “flat prosody.” “Lively prosody” was operationalized as prosody with great variations of intonation (baby birds’ story pitch rage: 412 Hz, dog’s story pitch range: 427 Hz) and changes of the timbre, according to who is talking each time (e.g., the cat while seeing the birds, or the dog while seeing the delicious sausages). “Flat prosody” was operationalized as prosody with relatively small variation of the intonation pattern (baby birds’ story pitch rage: 213 Hz, dog’s story pitch range: 207 Hz) and no changes of the timbre. The duration of the stories was almost the same for each of them in both conditions (approximately 75 s for the birds’ story and approximately 85 s for the dog’s story).

Each participant encountered both stories but each one in a different prosodic condition; no participant listened to the same story twice (once with lively and once with flat prosody) or the two stories with the same prosodic condition (both stories with lively or both stories with flat prosody). In particular, for every 5 children in each group, the following order/prosody condition combinations were made:

(a) Little birds-story with lively prosody – Dog-story with flat prosody
(b) Little birds-story with flat prosody – Dog-story with lively prosody
(c) Dog-story with flat prosody – Little birds-story with lively prosody
(d) Dog-story with lively prosody – Little birds-story with flat prosody

Participants’ comprehension skills were assessed with the use of ten questions per story. For each story, three questions addressed goal statements, five examined whether children understood the internal state of the characters which intrigued the initiating event or explain the characters’ reaction to events in the story, and the two final questions aimed at exploring higher aspects of ToM abilities, as they required children to make hypotheses about the internal state of the main characters.

### Procedure

Each participant was examined individually in a quiet room at the “The Down Syndrome Association of Greece” or at their home. Three colored envelopes were shown to each participant on the computer screen and they were asked to open one of them which included one of the stories. This procedure gave the participants the (false) belief that they were actually choosing the story, although the order and the story had been pre-arranged by the researchers. Then each participant listened to the story through headphones while viewing two pictures per slide on the computer screen. After listening to the whole story, the participants were presented again with all 6 pictures on the computer screen and were asked to retell the story to the examiner who was not present in the testing room. The examiner reminded the participants that she did not know the story. After the child’s retelling of the story, she was asked the comprehension questions. Each story was presented in a separate session. All experimental sessions were recorded using an OLYMPUS digital voice recorder (VN-8500pc).

### Data Analysis

Participants’ story retellings were transcribed and analyzed by two coders. The percentage agreement mean between the two coders was 92%. In the rest of the cases differences were discussed and changes were made where necessary. The adjusted ratings were then used for the statistical analyses.

The microstructure analysis measured the number of different content word types, the number of different function words, the MLU in words (because Greek is a high-inflectional language), as well as the number of verb-complement, adverbial, and relative clauses in each participant’s narrative.

For the macrostructure analysis MAIN (Gagarina et al., 2012) conventions were used. Each re-narration’s story structure was evaluated according to the following components: (1) setting component (reference of time and place), (2) internal state terms as initiating event, (3) goal, (4) attempt, (5) outcome and (6) internal state terms as reaction. Internal state terms, functioning as initiating events or as reactions to events or actions, consisted of both the ones which are related to ToM abilities, namely emotional (e.g., happy, sad, angry) and mental terms (e.g., believe, think, realize), and the ones which are not related to ToM abilities, such as perceptual (e.g., see, hear), physiological (e.g., thirsty, hungry), and communication (e.g., shout, say) terms (Gagarina et al., 2012; Tsimpli et al., 2016; Peristeri et al., 2017). Each story has been designed in such a way that the last five components occur three times per story. The first component is awarded with 2 points (1 for time and 1 for the place) and 1 point is awarded for each of the rest components. Therefore, the highest score of the use of internal state terms is 6 and the total maximum score is 17 points.

In some cases, however, participants could cover one component by using more than one internal state terms related to ToM in their re-narration or by using a ToM related internal term instead of a non-ToM internal state term. For example, instead of saying “The cat was *happy*” (one ToM related internal state term) or “The birds were *scared*” (one ToM related internal state term), they could say “The cat was *happy*, while the birds were *scared*” (two ToM related internal state terms) or instead of saying “mother *saw* the baby birds were hungry” (non-ToM related internal state term – perceptual term), they could say “mother *realized* the baby birds were hungry” (ToM related internal state term – mental term). For this reason, we calculated separately the ToM related internal state terms used by participants in their re-narrations, namely the number of unique lexical items expressing positive or negative emotion and mental verbs (e.g., think, wonder, believe, realize).

The comprehension questions were scored using one point for every correct answer to each one of the three questions about goal statements, five questions about the internal state of the characters, and two questions aiming at eliciting the participant’s prediction and explanation about the internal state of one of the main characters of the story (ToM abilities). Therefore, the maximum score for the comprehension questions was 10.

To ascertain differences among the groups in each task/variable, the data were analyzed using ANOVAs for each task/variable separately with the score of the task/variable as the dependent variable and Group as the independent variable. Main effects of Group were followed by *post hoc* Bonferroni tests to identify which groups differ from each other. To address differences between the groups for the prosody manipulation, two-way ANOVAs were conducted for each of the two macrostructure variables (story structure and comprehension questions) separately with the score of the variable as the dependent variable and Group as well as Prosody as independent variables.

## Research Hypotheses

Based on the previous literature we formulated the following hypotheses for the participants’ performance on micro- and macrostructure, comprehension questions, and prosodic manipulation:

1. ***Microstructure***: given that expressive vocabulary is indicative of the participants’ language abilities and that individuals’ with DS receptive, and not expressive, vocabulary abilities are reported to be relatively better than their morphosyntactic abilities, the DS group is expected to perform similarly to the expressive vocabulary-matched TD-EVT group and better than the TD-RCPM group, which consists of younger children who are matched on their non-verbal mental age.
2. ***Macrostructure***: the DS group is expected to produce re-narrations with better ***story structure*** compared to the ones produced by both control groups, due to their greater world-knowledge that relates to their age (Hudson and Shapiro, 1991; Cook and Guéraud, 2005; Finestack et al., 2013). Moreover, based on the findings of the study by Ashby et al. (2017), the DS group is expected to use fewer ***internal state terms***, as they are measured from the relevant components of the story structure conventions of MAIN, and fewer internal state terms related to ToM in their re-narrations than the two TD groups. Based on the Construction Hypothesis (Lindquist and Gendron, 2013), it could be predicted that all three groups would use few terms related to emotions, since all groups consist of preschool children or adults with mental age matched to that of preschool children and, thus, they would face difficulties in connecting emotional lexical terms with the character’s internal states. This tendency, however, is expected to be more pronounced for participants with DS, according to the results reported by Pochon and Declercq (2013, 2014) and Cebula et al. (2017).
3. ***Comprehension questions:*** the DS group is expected to be less accurate than both TD groups because they are reported to have poor comprehension abilities compared to TD children, even though their comprehension has been shown to be better than their production ability (e.g., Miolo et al., 2005).
4. ***Prosodic manipulation***: intense intonation and timbre is expected to improve TD children’s performance as far as the story structure and the accuracy on comprehension questions is concerned. This improvement is not expected to occur in the DS group performance because previous studies on children and adolescents with DS have reported difficulties in prosody comprehension (Pettinato and Verhoeven, 2009; Naess, 2016), whereas difficulties on speech production have also been related to impairments in prosody comprehension (Bray et al., 1995; Heselwood et al., 1995) (see also Perceptual Salience Approach; Echols and Newport, 1992).

## Results

### Baseline Tasks

Statistical analyses using ANOVAs with *post hoc* Bonferroni tests were first conducted on the groups’ scores on the baseline tasks in order to investigate possible group differences in non-verbal cognitive abilities, expressive and receptive vocabulary, sentence repetition, and phonological short-term memory. Table 2 shows the mean raw scores, standard deviations, and the range in each group in each one of the five baseline tasks, as well as the *p*-values for the comparisons between the three groups.

**TABLE 2:** Mean raw scores (SDs) and score range of the baseline tasks and *p*-values of the comparison between the DS group and the control groups.

	DS	TD-RCPM	TD-EVT
RCPM (max. = 36)			
Mean (*SD*)	12.75 (4.3)	14.80 (2.17)	22 (7.15)
Range	8–19	12–20	13–33
*p*-values		0.593	**<0.001**
EVT (max. = 50)			
Mean (*SD*)	27.45 (7.47)	22.55 (5.99)	32.50 (9.36)
Range	15–38	16–36	16–48
*p*-values		0.149	0.130
PPVT (max. = 173)			
Mean (*SD*)	91.80 (27.6)	53.15 (33.13)	109.25 (47.95)
Range	39–119	20–116	27–173
*p*-values		**0.005**	0.432
SRT (max. = 48)			
Mean (*SD*)	28.1 (12.97)	43.1 (3.62)	45.7 (4.17)
Range	10–46	32–48	32–48
*p*-values		**<0.001**	**<0.001**
FDR			
Mean (*SD*)	2.10 (2.9)	6.15 (3.15)	14.6 (7.84)
Range	0–12	1–12	4–30
*p*-values		**0.048**	**<0.001**

*DS, Down syndrome; TD-RCPM, Typically developing group matched to DS individuals on their age equivalent scores on the Raven’s Colored Progressive Matrices; TD-EVT, Typically developing group matched to DS individuals on their age equivalents scores on the Expressive Vocabulary Test; RCPM, Raven’s Colored Progressive Matrices Test; EVT, Expressive Vocabulary Test, PPVT = Peabody Picture Vocabulary Test, SRT = Sentence Repetition Test; FDR, Forward Digit Recall test; max., maximum score; SD, standard deviation; Bolded values indicate a statistically significant difference between the DS group and each of the two TD groups.*

Comparisons between the DS group and the TD-RCPM group showed that the DS group performed better than the TD-RCPM group on the PPVT task [*F*_(2,57)_ = 11.890, *p* = 0.005, $\eta_{\text{p}}^{2}$ = 0.294], but the opposite was the case for the SRT [*F*_(2,57)_ = 27.208, *p* < 0.001, $\eta_{\text{p}}^{2}$ = 0.488] and the FDR [*F*_(2,57)_ = 30.564, *p* = 0.048, $\eta_{\text{p}}^{2}$ = 0.517]. The two groups did not differ on the raw scores of the RCPM [*F*_(2,57)_ = 19.069, *p* = 0.593, $\eta_{\text{p}}^{2}$ = 0.401], since they were matched on this task, but they also did not differ on the EVT [*F*_(2,57)_ = 8.287, *p* = 0.149, $\eta_{\text{p}}^{2}$ = 0.225]. Comparisons between the DS group and the TD-EVT group showed that the EVT group performed better than the DS group on all tasks: RCPM [*F*_(2,57)_ = 19.069, *p* < 0.001, $\eta_{\text{p}}^{2}$ = 0.401], SRT [*F*_(2,57)_ = 27.208, *p* < 0.001, $\eta_{\text{p}}^{2}$ = 0.488], FDR [*F*_(2,57)_ = 30.564, *p* < 0.001, $\eta_{\text{p}}^{2}$ = 0.517]. The two groups did not differ on the raw scores of the EVT [*F*_(2,57)_ = 8.287, *p* = 0.30, $\eta_{\text{p}}^{2}$ = 0.225], since they were matched on this task. Finally, comparisons between the two control groups showed that the TD-EVT group performed significantly better than the TD-RCPM group on the RCPM [*F*_(2,57)_ = 19.069, *p* < 0.001, $\eta_{\text{p}}^{2}$ = 0.401], the EVT [*F*_(2,57)_ = 8.297, *p* < 0.001, $\eta_{\text{p}}^{2}$ = 0.225], the PPVT [*F*_(2,57)_ = 11.890, *p* < 0.001, $\eta_{\text{p}}^{2}$ = 0.294], and the FDR [*F*_(2,57)_ = 30.564, *p* < 0.001, $\eta_{\text{p}}^{2}$ = 0.517], but there was no statistical difference between the two groups on the SRT [*F*_(2,57)_ = 27.208, *p* = 0.992, $\eta_{\text{p}}^{2}$ = 0.488].

### Microstructure Analysis

Table 3 shows the results of the microstructure. The analyses comparing the DS group with the mental age control children showed that the DS group performed similarly to the TD-RCPM group in all microstructure measures [No of different content words: *F*_(2,117)_ = 23.913, p = 0.321, $\eta_{\text{p}}^{2}$ = 0.290; No of different function words: F_(2,117)_ = 24.478, *p* = 0.079, $\eta_{\text{p}}^{2}$ = 0.295; MLU in words: *F*_(2,117)_ = 14.337, *p* = 0.782, $\eta_{\text{p}}^{2}$ = 0.197; Adverbial clauses: *F*_(2,117)_ = 12.830, *p* = 1.000, $\eta_{\text{p}}^{2}$ = 0.180; Relative clauses: *F*_(2,117)_ = 1.833, *p* = 1.000, $\eta_{\text{p}}^{2}$ = 0.030], apart from the number of complement clauses [*F*_(2,117)_ = 7.586, *p* = 0.031, $\eta_{\text{p}}^{2}$ = 0.115] (see Table 3), for which the TD-RCPM group produced more complement clauses than the DS group.

**TABLE 3:** Mean raw scores (SDs) and score range of each microstructure variable and *p*-values of the comparison between the DS group and the control groups.

	DS	TD-RCPM	TD-EVT
No of different content words			
Mean (*SD*)	14.98 (4.52)	16.80 (4.23)	22.43 (6.11)
Range	8–27	9–28	12–32
*p*-values		0.321	**<0.001**
Noof different function words			
Mean (*SD*)	3.23 (1.63)	4.20 (1.88)	6.20 (2.26)
Range	0–7	1–8	2–13
*p*-values		0.79	**<0.001**
MLU in words			
Mean (*SD*)	5.47 (1.06)	5.17 (0.87)	6.53 (1.54)
Range	4–6.75	3.14–7.67	4.43–9.28
*p*-values		0.782	**<0.001**
Adverbial clauses			
Mean (*SD*)	0.45 (0.82)	0.53 (0.75)	1.55 (1.52)
Range	0–4	0–3	0–5
*p*-values		1.000	**0.001**
Relative clauses			
Mean (*SD*)	0.13 (0.46)	0.23 (0.48)	0.35 (0.62)
Range	0–2	0–2	0–2
*p*-values		1.000	0.175
Complement clauses			
Mean (*SD*)	0.85 (1.21)	1.5 (1.06)	1.8 (1.07)
Range	0–5	0–4	0–4
*p*-values		**0.031**	**0.001**

*DS, Down syndrome; TD-RCPM, Typically developing group matched to DS individuals on their age equivalent scores on the Raven’s Colored Progressive Matrices; TD-EVT, Typically developing group matched to DS individuals on their age equivalents scores on the Expressive Vocabulary Test; SD, standard deviation; Bolded values indicate a statistically significant difference between the DS group and each of the two TD groups.*

Comparison between the DS group and the EVT group revealed that the TD-EVT group performed significantly better than the DS group in all microstructure variables [No of different content words: *F*_(2,117)_ = 23.913, *p* < 0.001, $\eta_{\text{p}}^{2}$ = 0.290; No of different function words: *F*_(2,117)_ = 24.478, *p* < 0.001, $\eta_{\text{p}}^{2}$ = 0.295; MLU in words: *F*_(2,117)_ = 14.337, *p* < 0.001, $\eta_{\text{p}}^{2}$ = 0.197; Adverbial clauses: *F*_(2,117)_ = 12.830, *p* < 0.001, $\eta_{\text{p}}^{2}$ = 0.180; Complement clauses: *F*_(2,117)_ = 7.586, *p* = 0.001, $\eta_{\text{p}}^{2}$ = 0.115], apart from the use of Relative clauses [*F*_(2,117)_ = 1.833, *p* = 0.175, η*p*^2^ = 0.030].

Moreover, comparisons between the two control groups showed that the TD-EVT group performed significantly better in all microstructure variables compared to the TD-RCPM group [No of different content words: *F*_(2,117)_ = 23.913, *p* < 0.001, $\eta_{\text{p}}^{2}$ = 0.290; No of different function words: *F*_(2,117)_ = 24.478, *p* < 0.001, $\eta_{\text{p}}^{2}$ = 0.295; MLU in words: *F*_(2,117)_ = 14.337, *p* < 0.001, $\eta_{\text{p}}^{2}$ = 0.197; Adverbial clauses: *F*_(2,117)_ = 12.830, *p* < 0.001, $\eta_{\text{p}}^{2}$ = 0.180; Complement clauses: *F*_(2,117)_ = 7.586, *p* = 0.694, $\eta_{\text{p}}^{2}$ = 0.115], apart from the use of Relative clauses. [*F*_(2,117)_ = 1.833, *p* = 0.872, $\eta_{\text{p}}^{2}$ = 0.030].

Finally, as the groups were found to differ in the Sentence Repetition Task and the Forward Digit Recall test, scores on microstructure variables were also analyzed with Sentence Repetition and Forward Digit Recall as covariates, in order to examine whether microstructure scores per measure changed as a result. In particular, ANCOVA analyses were performed with the group as the between-subjects variable, Sentence Repetition Task and Forward Digit Recall test as covariates and each of the microstructure variables as the dependent variable. Both covariates were found to be unrelated to all microstructure variables.

### Macrostructure Analysis

Table 4 shows the results of the macrostructure. The analyses comparing the DS group with the mental age control children revealed that the TD-RCPM group performed significantly better than the DS group on the use of internal state terms in the story structure form [*F*_(2,177)_ = 8.370, *p* = 0.049, $\eta_{\text{p}}^{2}$ = 0.125] and the use of ToM related internal state terms in their re-narrations [*F*_(2,177)_ = 8.022, *p* = 0.027, $\eta_{\text{p}}^{2}$ = 0.121]. In contrast, the DS group performed significant better than the TD-RCPM group on the questions related to the ToM [*F*_(2,177)_ = 10.125, *p* = 0.025, $\eta_{\text{p}}^{2}$ = 0.148]. The two groups did not differ from each other on story structure [*F*_(2,177)_ = 16.520, *p* = 0.131, $\eta_{\text{p}}^{2}$ = 0.220], comprehension questions [*F*_(2,177)_ = 5.819, *p* = 1.000, $\eta_{\text{p}}^{2}$ = 0.090] and on correctly reporting characters’ internal state terms in comprehension questions [*F*_(2,177)_ = 5.107, *p* = 1.000, $\eta_{\text{p}}^{2}$ = 0.054].

**TABLE 4:** Mean raw scores (SDs) and score range of each macrostructure variable and *p*-values of the comparison between the DS group and the control groups.

	DS	TD-RCPM	TD-EVT
Story Structure (max. = 17)			
Mean (*SD*)	4.43 (1.5)	5.23 (1.73)	6.65 (1.99)
Range	2–8	2–10	3–10
*p*-values		0.131	**<0.001**
IST in story structure (max. = 6)			
Mean (*SD*)	0.55 (0.75)	1.08 (1.16)	1.43 (0.93)
Range	0–2	0–4	0–4
*p*-values		**0.049**	**<0.001**
ToM in retellings			
Mean (*SD*)	0.43 (0.68)	1.00 (0.96)	1.28 (1.20)
Range	0–3	0–3	0–6
*p*-values		**0.027**	**<0.001**
Comprehension questions			
(max. = 10)			
Mean (*SD*)	6.05 (2.68)	6.53 (1.78)	7.70 (2.13)
Range	2–10	3–10	3–10
*p*-values		1.000	**0.004**
IST in comprehension questions			
(max. = 6)			
Mean (*SD*)	3.20 (2)	3.30 (1.62)	4.35 (1.7)
Range	1–6	1–6	1–6
*p*-values		1.000	0.061
ToM in comprehension questions			
(max. = 2)			
Mean (*SD*)	0.98(0.92)	0.45 (0.78)	1.33 (0.92)
Range	0–2	0–2	0–2
*p*-values		**0.025**	0.229

*DS, Down syndrome; TD-RCPM, Typically developing group matched to DS individuals on their age equivalent scores on the Raven’s Colored Progressive Matrices; TD-EVT, Typically developing group matched to DS individuals on their age equivalents scores on the Expressive Vocabulary Test; IST, Internal State Terms; ToM, Theory of Mind; SD, standard deviation; Bolded values indicate a statistically significant difference between the DS group and each of the two TD groups.*

The analyses comparing the DS group with the vocabulary control children showed that the TD-EVT group performed significantly better than the DS group on story structure [*F*_(2,177)_ = 16.520, *p* < 0.001, $\eta_{\text{p}}^{2}$ = 0.220], internal state terms in story structure [*F*_(2,177)_ = 8.370, *p* < 0.001, $\eta_{\text{p}}^{2}$ = 0.125], ToM in retellings [*F*_(2,177)_ = 8.022, *p* < 0.001, $\eta_{\text{p}}^{2}$ = 0.121] and on comprehension questions [*F*_(2,177)_ = 5.819, *p* = 0.004, $\eta_{\text{p}}^{2}$ = 0.090]. The two groups did not differ from each other on the comprehension questions related to characters’ internal state terms [*F*_(2,177)_ = 3.339, *p* = 0.061, $\eta_{\text{p}}^{2}$ = 0.054] and on the comprehension questions related to the ToM [*F*_(2,177)_ = 10.125, *p* = 0.229, $\eta_{\text{p}}^{2}$ = 0.148].

Furthermore, comparisons between the TD-RCPM and the TD-EVT group revealed that the TD-EVT group performed significantly better than the TD-RCPM group on story structure [*F*_(2,177)_ = 16.520, *p* = 0.001, $\eta_{\text{p}}^{2}$ = 0.220] and on the questions related to the ToM [*F*_(2,177)_ = 10.125, *p* < 0.001, $\eta_{\text{p}}^{2}$ = 0.148]. No statistical significant differences were found between the two groups on the use of internal state terms in the story structure [*F*_(2,177)_ = 8.370, *p* = 0.320, $\eta_{\text{p}}^{2}$ = 0.125], the use of ToM related internal state terms in participants’ re-narrations [*F*_(2,177)_ = 8.022, *p* = 0.620, $\eta_{\text{p}}^{2}$ = 0.121], comprehension questions [F_(2,177)_ = 5.819, p = 0.060, $\eta_{\text{p}}^{2}$ = 0.090] and on correctly reporting characters’ internal state terms in comprehension questions [*F*_(2,177)_ = 5.107, *p* = 0.113, $\eta_{\text{p}}^{2}$ = 0.054].

Lastly, ANCOVA analyses were performed with group as the between-subjects variable, Sentence Repetition Task and Forward Digit Recall test as covariates and each of the microstructure variables as the dependent variable. Results revealed no relation between any of the two covariates and each of the microstructure variables.

## The Role of Prosody on Macrostructural Performance

Table 5 shows the results of the prosody manipulation on the children’s performance on the macrostructure. The analyses for story structure demonstrated a main effect of Group [*F*_(2,114)_ = 17.638, *p* < 0.001, $\eta_{\text{p}}^{2}$ = 0.236] and Prosody [*F*_(2,114)_ = 12.956, *p* < 0.001, $\eta_{\text{p}}^{2}$ = 0.102], but no interaction between Group and Prosody [*F*_(2,114)_ = 0.145, *p* = 0.865, $\eta_{\text{p}}^{2}$ = 0.003]. All three groups performed significantly better when prosody was “lively,” compared to their own performance when prosody was “flat.” The TD-EVT group performed better on both prosodic versions compared to each of the other two groups (TD-EVT vs. DS: *p* < 0.001; TD-EVT vs. TD-RCPM: *p* = 0.001).

**TABLE 5:** Mean scores (SDs) and range of correct responses of macrostructure variables with lively and flat prosody.

	DS		TD-RCPM		TD-EVT	
	Lively prosody	Flat prosody	Lively prosody	Flat prosody	Lively prosody	Flat prosody
Story structure (max. = 17)						
Mean (*SD*)	4.95 (1.19)	3.90 (1.62)	5.86 (1.9)	4.53 (1.22)	7.10 (1.9)	6.16 (2.04)
Range	4–7	2–7	3–10	2–6	4–10	4–10
Comprehension Questions (max. = 10)						
Mean (*SD*)	6.40 (2.64)	5.70 (2.74)	7.05 (1.75)	5.95 (1.68)	8.29 (1.98)	7.05 (2.15)
Range	2–10	3–10	5–10	3–10	5–10	3–10

*DS, Down syndrome; TD-RCPM, Typically developing group matched to DS individuals on their age equivalent scores on the Raven’s Colored Progressive Matrices; TD-EVT, Typically developing group matched to DS individuals on their age equivalents scores on the Expressive Vocabulary Test; SD, standard deviation; max., maximum score.*

Similar findings were revealed for the comprehension questions. There was a main effect of Group [*F*_(2,114)_ = 5.807, *p* = 0.004, $\eta_{\text{p}}^{2}$ = 0.092] and Prosody [*F*_(2,114)_ = 6.368, *p* = 0.013, $\eta_{\text{p}}^{2}$ = 0.053], but no interaction effect between Group and Prosody [*F*_(2,114)_ = 0.160, *p* = 0.852, $\eta_{\text{p}}^{2}$ = 0.003]. All three groups performed significantly better when prosody was “lively,” compared to their own performance when prosody was “flat.” The TD-EVT performed better compared to the two groups both when prosody was “lively” or “flat” (TD-EVT vs. DS: *p* < 0.001; TD-EVT vs. TD-RCPM: *p* = 0.002).

## Discussion

The aim of this study was to investigate the language and socio-cognitive abilities of adults with DS, an understudied group compared to children and adolescents with DS. To address this aim, we used a narrative re-telling task that provides a wealth of information about the participants’ morphosyntactic production through the microstructure analysis, their socio-cognitive abilities through the macrostructure analysis, and their comprehension abilities through comprehension questions. An additional prosodic manipulation enabled us to address whether or not lively prosody can enhance their comprehension. Thus, this narrative task enabled us to conduct a systematic examination of their language and socio-cognitive abilities.

Twenty adults with DS and 40 TD control children completed a battery of baseline and experimental tasks. Half of the TD children were matched to the DS group on mental age and the other half were matched on expressive vocabulary. This enabled to address whether the adults with DS perform similarly to TD children with the same mental age or expressive vocabulary. The baseline tasks consisted of the RCPM (Raven et al., 2008), the EVT (Vogindroukas et al., 2009), the PPVT (Simos et al., 2011), the SRT (Stavrakaki and Tsimpli, 2000), and a FDR (Paraskevopoulos et al., 1996). Interestingly, no statistically significant difference was found on the EVT between the group with DS and the RCPM group [for a similar pattern see also Laws and Bishop (2003)]. The baseline tasks revealed that the DS group demonstrated statistically significant poor performance on the SRT and the FDR compared to both groups of TD children, whereas adults’ with DS performance on the PPVT was significantly better compared to the TD-RCPM group. Given that the SRT and the FDR explore morphosyntactic skills and phonological short term/working memory, whereas the PPVT examines receptive vocabulary abilities, the results are in line with the characteristics usually attributed to individuals with DS, namely impaired morphosyntactic abilities, poor phonological memory and relatively good vocabulary knowledge which is more apparent in comprehension than in production (e.g., Abbeduto et al., 2003, 2007; Phillips et al., 2014). The results from ANCOVAs revealed that scores on the SRT and the FDR did not relate to the performance on any micro- or macrostructural variables.

The experimental task consisted of two stories. Each participant listened to the two stories, one produced with “flat” and one with “lively” prosody, while seeing six relevant pictures and then they had to re-narrate the story with the use of the wordless pictures. Comprehension questions followed each narration.

The first hypothesis regarded the participants’ performance on **microstructure**. We hypothesized that the DS group will perform similarly to the vocabulary-matched TD-EVT group and better than the TD-RCPM group, which consists of younger children who are matched on their non-verbal mental age. This was based on the idea that expressive vocabulary is indicative of the participants’ general language abilities, and therefore, if we match groups on vocabulary, they would perform similarly also on morphosyntax.

The results did not support this hypothesis. The adults with DS performed less well than the vocabulary matched TD-EVT group in every microstructure variable, apart from the use of Relative clauses, in which the difference did not reach significance, since every group used a very small number of Relative clauses. This pattern is in line with the descriptions of the phenotype of individuals with DS, according to which vocabulary knowledge is their strength, i.e., the aspect in which their skills exceed their general performance, and thus, their performance on vocabulary tasks is not representative of their performance on other tasks. One could support, though, that these findings contradict the outcomes of empirical studies (e.g., Miller, 1991; Ukrainetz and Blomquist, 2002; Lee, 2011; DeThrone, unpublished) and theoretical accounts (Bates and Goodman, 1997, 1999, 2001) suggesting a link between lexical and morphosyntactic abilities since the DS group performed less well in all morphosyntactic variables compared to the group matched to individuals with DS on expressive vocabulary. On the other hand, the fact that participants’ with DS re-narrations contained both significantly less content and function words and less MLU and specific morphosyntactic structures do not allow us to reach conclusive assumptions regarding the existence or not of a single learning mechanism governing both lexical and morphosyntactic development. The DS group performed similarly to the non-verbal mental age matched TD-RCPM group in all variables apart from the use of Complement clauses, in which the DS group had significantly lower scores than each of the two TD groups. These findings did not support our hypothesis that the DS group will perform better than the TD-RCPM group on the microstructure variables. These results are, however, in line with the findings reported by previous studies (e.g., Miles et al., 2006; Keller-Bell and Abbeduto, 2007), in which the DS group was reported to perform similarly to the TD group matched on non-verbal mental age. The performance of the DS group on Complement clauses is particularly low and requires further discussion for the reasons for their very low performance. Verb-Complement clauses are clauses selected by the verb and consequently both lexical and morphosyntactic knowledge is necessary for their correct use (e.g., Haegeman, 2006). In contrast, Adverbial and Relative clauses are not selected by the verb, but they are mainly used by the narrators for semantic purposes, i.e., in order for coherence to be established (Vieu et al., 2005). The restricted use of Complement clauses by the DS group confirms their poor morphosyntactic abilities and demonstrates that when both lexical and morphosyntactic knowledge is required, adults with DS may perform less well than even mental age matched controls.

The second hypothesis regarded the participants’ performance on ***macrostructure***. We hypothesized that the DS group will produce re-narrations with better story structure compared to the ones produced by both control groups. This was based on the idea that their greater world-knowledge that relates to their age can support their performance on story structure. Moreover, based on the findings reported by Ashby et al. (2017), according to which participants with DS used less inferential language compared to their TD counterparts matched on non-verbal mental age, we hypothesized that the DS group would use fewer ToM references and number of internal state terms compared both to the TD-RCPM of the present study and to the TD-EVT group, which consists of older children with, consequently, higher non-verbal mental age. The results did not support the first part of our hypothesis. The DS group performed less well compared to the TD-EVT group and similarly to the TD-RCPM group. This suggests that greater world-knowledge due to greater age does not necessarily translate to a better performance on story structure.

The pattern of similar performance to the TD-RCPM group can be further discussed in relation to the study by Finestack et al. (2012). Finestack et al. (2012) included one control group matched on non-verbal mental age and one matched on MLU and found that the DS group performed better in introducing characters and describing the story-events compared to their TD group matched on non-verbal mental age, but no difference in comparison to the MLU matched TD group. The discrepancy between the findings of Finestack et al. (2012) and our study are likely to relate to the different characteristics of the control groups in the two studies. In our study, the TD-RCPM group was matched to the DS group on non-verbal mental age but results on the microstructure demonstrate that they had also similar MLU (for a similar pattern, see also Keller-Bell and Abbeduto, 2007). Therefore, the findings regarding the comparison between the group with DS and the TD-RCPM are in line with the ones reported on Finestack et al. (2012) study when considering the groups’ MLU performance. Finestack et al. (2012) commented that their results suggest the existence of a closer association between macrostructural and microstructural language ability than between macrostructural and non-verbal mental ability in individuals with DS.

The results of internal state terms and the number of ToM references support the hypothesis that the DS group will use fewer ToM references and number of internal state terms than the two TD groups. Both control groups performed better than the group of adults with DS. These findings differ from the results by Ashby et al. (2017) that showed similar performance when the DS group was matched to the control group on MLU in that in the present study the TD-RCPM matched to the DS group both on non-verbal mental age and MLU, but still adults’ with DS performance was poorer compared to both TD groups. Ashby et al. (2017) questioned whether individuals’ with DS poor performance reflected their inability to recognize characters’ internal states or their poor morphosyntactic abilities which constrain them to use more complex structures, as the ones needed in describing someone’s internal state. In order to answer this question, we should reconsider the individuals’ with DS performance on microstructure variables and examine whether there is a connection between any of them and the description of main characters’ feelings and beliefs. As we have seen above, the group with DS performed significantly worse than both controls groups on the use of Complement clauses. Complement clauses are selected as complements of a verb in the higher clause. Mental verbs (e.g., believe, think, realize, remember and wonder), which together with emotional terms are considered to be the internal state terms connected to ToM, always need a complement clause. This suggests that adults with DS have not mastered yet the complement structures which permit the representation of embedded sentences about internal states, and thus, they do not have the means to express other people’s feelings, beliefs and thoughts. The connection between the use of complement clauses and the use of internals state terms (related or non-related to ToM) can be also seen on both controls groups, since they performed better compared to individuals with DS on both the use of complement clauses and the use of internals state terms. In other words, there seems to be a close connection between participants’ performance on morphosyntax, and especially the use of complement clauses, and their performance on the use of internal state terms, especially the ones connected to ToM (de Villiers and de Villiers, 2000, 2003, 2009; de Villiers, 2005; Schick et al., 2007). Another way to explore whether DS group’s poor performance on describing main characters’ feelings and beliefs is due to morphosyntax and not due to their inability in recognizing them, is to examine their story comprehension with the use of Comprehension questions.

The third hypothesis regarded the participants’ performance on ***comprehension questions***. We hypothesized that the DS group will be less accurate than both TD groups because they are reported to have poor comprehension abilities compared to TD children, although their comprehension has been shown to be better than their production ability (e.g., Miolo et al., 2005). The results showed that the DS group performed similarly to the TD-RCPM group on comprehension questions and significantly worse compared to the TD-EVT group. These findings partially support the hypothesis and are in line with the overall better performance of the TD-EVT group compared to the other groups, as well as the similar performance of the DS and TD-RCPM group on most variables. A closer look at the results shows that the DS group performs similarly to each of the two TD groups on the questions related to the internal states of the main characters. Similarly, as far as accuracy on questions related to ToM is concerned, the DS group performed similarly to the TD-EVT group and outperformed the TD-RCPM group. This suggests that individuals with DS understood the main characters’ internal states in the same way as the TD children did and recognized the ToM internal states better than the TD-RCPM group. In other words, it seems that individuals with DS were able to understand the main characters’ feelings, beliefs and intentions, based on the prototypical narrations, the relevant pictures, and their world knowledge, but they were unable to present/explain them in their re-narrations. For example, they found difficulty in using mental terms, such as the verb “want” with its complement clause (e.g., “Mother *wanted to find* food for her baby birds”), but when they were asked “Why did the mother bird fly away?” they could say “*To find* food for her babies” (adverbial clause). Similarly, they used less emotional terms, such as “happy,” “sad,” but when they were asked, for example, “How does the boy feel,” while the experimenter pointing to the relevant picture, they responded correctly (happy) and they could also explain why (e.g., “Because he took his balloon back”). They were even able to answer to more hypothetical questions demanding ToM abilities, such as “Imagine that the boy sees the dog (eating his sausages). How do you think that he would feel?” and then “Why do you think that he would feel … (whatever word the participant used in the previous question e.g., sad or angry). This supports our claim that individuals’ with DS renarrations did not contain enough internal state terms (related or not related to ToM), not because of their inability to recognize them, but due to their poor morphosyntactic abilities, which did not allow them to find the proper means to express the main characters’ internal states. As for the Construction Hypothesis (Lindquist and Gendron, 2013), according to which the use of internal state terms is related to the understanding of the exact meaning of the words referring to emotions, it can account for the performance of the two control groups in that they performed similarly regarding the use of internal state terms in their re-narrations (production) and in comprehension questions, but not for the performance of the group with DS. In particular, it seems that individuals with DS understood the meaning of the words expressing emotions, as their performance on comprehension questions reveals, even though they do not use them in their re-narrations. The different patter attested on Pochon and Declercq’s (2013, 2014) and Cebula et al.’s (2017) studies and ours can be attributed to the fact that, in contrast to their studies, our participants with DS are adults and not children, and we used narrations with pictures and not a photo-matching emotion label task. Therefore, it could be suggested that our participants’ with DS world-knowledge helped them to correctly match the emotions to words, while the context of the narratives helped them to better understand the characters’ mental state, even though they did not report them in their re-narrations.

The fourth hypothesis regarded the role of ***prosody*** in the participants’ performance on story structure and comprehension questions. We hypothesized that intense intonation and timbre would improve TD children’s performance but not the performance of the DS group because previous studies on children and adolescents with DS have reported difficulties in prosody comprehension (e.g., Pettinato and Verhoeven, 2009; Naess, 2016). This hypothesis was partially supported by the data. The results showed that all three groups performed significantly better on Story structure and Comprehension questions when prosody was “lively” compared to their own performance when prosody was “flat.” Thus, it might be the case that individuals’ with DS difficulties in comprehending prosody are mainly evident in word-level units of speech, like on word stress (Pettinato and Verhoeven, 2009) and word rhyming (Naess, 2016). When bigger chunks are made salient, such as sentences and small passages, then individuals with DS manage to better comprehend the global concept of these parts of speech. This claim can be considered to be in line with the outcomes of several studies, according to which people with DS are reported to favor global information at the expense of local processing (e.g., Porter and Coltheart, 2006). Moreover, it has been claimed that the Perceptual Salience Approach can be also applied in broader units, such as in the identification of syntactic structures (e.g., Morgan and Newport, 1981). In this respect, prosody, such as intonation and timbre used in the present study, can be considered to make sentences, phrases, and small passages more salient. On the other hand, the examination of different prosodic cues (stress and rhyme vs. intonation and timbre) can account for the different results found in previous studies and the present one. The finding that prosody can assist individuals’ with DS comprehension is of great importance, since apart from pictures (Miles et al., 2006), prosody seems to be another way to support their comprehension.

## Conclusion

The aim of the present study was to investigate the language and socio-cognitive abilities of an understudied group, adults with DS, and to offer a clearer and more complete view of their skills and impairments as far as language, cognitive ability, and prosody is concerned. To this end, a story-retelling task was given to 20 adults with DS, 20 TD children matched on on-verbal mental age (TD-RCPM) and 20 TD children matched on expressive vocabulary (TD-EVT).

The results revealed that overall the DS adults performed similarly to the TD-RCPM group and less well that the TD-EVT group which outperformed both groups in almost all measures. Moreover, the DS group performed less well than the TD-RCPM group on the use of Complement clauses and the use of internal terms (related or not related to ToM) in their re-narrations. However, given that mental verbs select complement clauses, in combination with the findings that the DS group showed similar accuracy in Comprehension questions with the TD-RCPM group and better than the TD-RCPM group on the questions regarding internal state terms related to ToM, suggests that adults with DS face difficulties not with the recognition of other people’s internal states, but with the morphosyntactic structures needed in describing someone’s internal state. The manipulation of prosody showed that “lively” prosody improved the performance of adults with DS in remembering the story (story retelling) and in answering the comprehension questions. This suggests that “lively” prosody helped them comprehend the stories and exceed their language and cognitive plateau (Cuskelly et al., 2016; Witecy and Penke, 2017).

To conclude, this is the first study that investigates systematically the language, socio-cognitive, and prosodic abilities of adults with DS using a narrative retelling task. The results revealed that adults with DS face difficulties with morphosyntax; this can constrain them to express people’s internal states, but they are able to use prosody effectively to enhance their comprehension in a similar way as TD controls. This has important implications for intervention programmes designed for individuals with DS. Intervention programs based on prosody could help adults with DS improve their language and cognitive skills. More research is needed in order to reach safe conclusions regarding the extent to which prosody can enhance the adults’ with DS comprehension abilities and to shed light into the relationship between morphosyntactic and socio-cognitive abilities of adults with DS.

## Data Availability Statement

The datasets generated for this study are available on request to the corresponding author.

## Ethics Statement

This study was reviewed by the University of Reading School of Psychology and Clinical Language Sciences’ Research Ethics Committee and was given a favorable ethical opinion for conduct (2019-193-TM). Written informed consent to participate in this study was provided by the participants’ legal guardian/next of kin.

## Author Contributions

MM conceived the idea, designed the experiments, collected and analyzed the data, and wrote the manuscript. AN contributed to the data collection. TM contributed to the design of the experiments, to the interpretation of the results, and to the writing of the manuscript. All authors contributed to the article and approved the submitted version.

## Conflict of Interest

The authors declare that the research was conducted in the absence of any commercial or financial relationships that could be construed as a potential conflict of interest. The reviewer VS declared a shared affiliation, with no collaboration, with one of the authors, TM, to the handling editor at the time of the review.
